# Breast Cancer Tissue Explants: An Approach to Develop Personalized Therapy in Public Health Services

**DOI:** 10.3390/jpm13101521

**Published:** 2023-10-23

**Authors:** Pilar Carranza-Rosales, Daniel Valencia-Mercado, Olga Esquivel-Hernández, Manuel Ismael González-Geroniz, José Inocente Bañuelos-García, Ana Lilia Castruita-Ávila, Mario Alberto Sánchez-Prieto, Ezequiel Viveros-Valdez, Javier Morán-Martínez, Isaías Balderas-Rentería, Nancy Elena Guzmán-Delgado, Irma Edith Carranza-Torres

**Affiliations:** 1 Centro de Investigación Biomédica del Noreste, Instituto Mexicano del Seguro Social, Calle Jesús Dionisio González # 501, Col. Independencia, Monterrey 64720, NL, Mexico; maria.carranza@imss.gob.mx; 2 Unidad Médica de Alta Especialidad, Hospital de Ginecología y Obstetricia No. 23, Instituto Mexicano del Seguro Social, Avenida Constitución y Félix U, Gómez s/n, Colonia Centro, Monterrey 64000, NL, Mexico; daniel.valenciam@imss.gob.mx (D.V.-M.); oesh10@gmail.com (O.E.-H.); manuel.gonzalezg@imss.gob.mx (M.I.G.-G.); drjibg@hotmail.com (J.I.B.-G.); 3 Unidad Médica de Alta Especialidad, Hospital de Especialidades No. 25, Instituto Mexicano del Seguro Social, Av Fidel Velázquez s/n, Mitras Nte., Monterrey 64180, NL, Mexico; anacastruita@hotmail.com (A.L.C.-Á.); drmasp87@gmail.com (M.A.S.-P.); 4 Facultad de Ciencias Biológicas, Universidad Autónoma de Nuevo León, Av. Pedro de Alba s/n, San Nicolás de los Garza 66450, NL, Mexico; jose.viverosvld@uanl.edu.mx; 5 Departamento de Biología Celular y Ultraestructura, Facultad de Medicina, Universidad Autónoma de Coahuila, Av. Morelos 900-Oriente, Primera de Cobián Centro, Torreón 27000, CH, Mexico; javiermoranmartinez@uadec.edu.mx; 6 Facultad de Ciencias Químicas, Universidad Autónoma de Nuevo León, Av. Pedro de Alba s/n, San Nicolás de los Garza 66450, NL, Mexico; isaias.balderasrn@uanl.edu.mx; 7 Unidad Médica de Alta Especialidad, Hospital de Cardiología No. 34, Instituto Mexicano del Seguro Social, Av. Lincoln S/N, Col. Valle Verde 2do. Sector, Monterrey 64360, NL, Mexico

**Keywords:** breast cancer, tissue slices, explants, ex vivo, chemotherapy, treatment response

## Abstract

Breast cancer is one of the main causes of death worldwide. Lately, there is great interest in developing methods that assess individual sensitivity and/or resistance of tumors to antineoplastics to provide personalized therapy for patients. In this study we used organotypic culture of human breast tumor slices to predict the experimental effect of antineoplastics on the viability of tumoral tissue. Samples of breast tumor were taken from 27 patients with clinically advanced breast cancer; slices were obtained and incubated separately for 48 h with paclitaxel, docetaxel, epirubicin, 5-fluorouracil, cyclophosphamide, and cell culture media (control). We determined an experimental tumor sensitivity/resistance (S/R) profile by evaluating tissue viability using the Alamar Blue^®^ metabolic test, and by structural viability (histopathological analyses, necrosis, and inflammation). These parameters were related to immunohistochemical expression of the estrogen receptor, progesterone receptor, and human epidermal growth factor receptor 2. The predominant histological type found was infiltrating ductal carcinoma (85.2%), followed by lobular carcinoma (7.4%) and mixed carcinoma (7.4%). Experimental drug resistance was related to positive hormone receptor status in 83% of samples treated with cyclophosphamide (*p* = 0.027). Results suggest that the tumor S/R profile can help to predict personalized therapy or optimize chemotherapeutic treatments in breast cancer.

## 1. Introduction

Breast cancer is the most common cancer in women, with the highest morbidity and mortality rates worldwide [1]. In Mexico, breast cancer has been ranked first place in mortality for all types of cancer in women since 2006 [2,3]. Although there are new therapeutic strategies, the progression of the disease and deaths are still among the main problems resulting from each patient’s acquired and intrinsic resistance, and it has not yet been possible to determine which chemotherapy drugs could be the most effective individualized for each case. Due to its socioeconomic impact, cancer treatment has begun to evolve toward a personalized approach based on the complexity of the patient and tumor molecular profile [4,5,6,7]. Several molecular biomarkers have been used to develop targeted therapeutic strategies; however, in most cases, these biomarkers cannot predict the individual response to chemotherapy [8]. Personalized medicine analyzes clinical, genetic, and genomic information, as well as the environment surroundings, in order to apply the most individualized treatment possible [9].

Most of the research and clinical trials for breast cancer treatment are based on the use of tumoral cell lines and animal models for in vitro and in vivo assays, respectively [10,11]. However, primary cultures or established cell lines do not fully reflect the complex tissue architecture of the tumor, limiting their predictive value [12,13]. For in vivo experiments, cell-derived xenografts and patient-derived xenografts (PDX) are the most widely used preclinical models. These systems use immunodeficient and/or genetically modified mice that have been altered with primary cultures of human cancer cells or fragments of human tumors that are grafted to preserve the basic characteristics of the primary tumors [14,15,16,17]. One major issue is the murine and non-human stroma origin. Furthermore, the host–xenograft interactions that take place under the skin may be different from those that occur in the tissue where the tumor originates [18,19], and the genetic background of each animal strain has been extremely altered [20]. To reduce these disadvantages, some recent PDX models have been designed using humanized mice (carried out by transplantation of peripheral blood mononuclear cells or hematopoietic stem cells into immunodeficient mice); they allow the interaction between the human immune system and human tumor growth, improving the physiologically human-relevant scenery [21,22]. 

Preclinical three-dimensional (3D) models, such as spheroids, organoids, organ-on-a-chip, perfusion bioreactors, and patient-derived organoids, are extensively used in the investigation of biomarkers or techniques that allow the response to chemotherapy to be predicted in patients with cancer, or in the search for new anticancer drugs. However, despite their efficacy, currently very few 3D models have been incorporated to routinely investigate new drugs and anticancer activity and to be more representative of the in vivo condition. This is possibly because these models are still unable to recapitulate the true cellular richness, tumor microenvironment (TME), the spatial complexity of the original tissue, or accurately predict the drug effects. In addition, reproducibility, economical cost, and limited performance are some of the obstacles that prevent their routine use [23].

Because of the important role of the TME in the response of tumoral cells to cytotoxic therapy, it is necessary that preclinical models consider not only the complexity of the tumor but also intratumoral heterogeneity [24]. In fact, the same tumor can contain more than 10 different intratumoral regions, each expressing different gene profiles [25]. Preclinical models that make it possible to identify individual differences in the tumor response to different therapeutic drugs could help design specific regimens for each patient, which increases the probability of achieving the most favorable response [26]. According to Conde et al. [5], to study tumor behavior, it is necessary to maintain or reconstitute a microenvironment like that of the tumor in situ. Although their clinical utility is still being studied, ex vivo human organotypic culture techniques fill the gap for this requirement and could represent a rapid animal-free preclinical screening platform to evaluate different therapeutic regimens, stratify patients who may potentially respond to chemotherapy agents, and prevent other patients from experiencing their adverse toxic effects [7,11]. 

One of the models used for this purpose is the culture of precision-cut tumor tissue slices, an ex vivo 3D system that closely resembles the in vivo TME [27]. Precision-cut tissue slices represent the complexity of the intact organ; facilitate histological evaluation as an alternative or biochemical tests; permit cocultured slices derived from different organs from the same donor; and facilitate the investigation of regional toxicity, studies on metabolism, and efficient use of human tissue [28]. For these reasons, tissue slices are considered “miniorgans” because they contain practically all cellular types from the tissue under study and preserve the histological and 3D structure of the organ from which they are obtained, while maintaining their intercellular and extracellular interactions and elements of the cellular matrix, and interestingly, they preserve their metabolic capacity [29,30,31,32]. In addition, the in vitro concentrations that reproduce the injury seen in vivo often correlate with the in vivo drug plasma exposure levels associated with toxicity [33]. Recently, many investigators have used this ex vivo 3D system since it preserves the tissue architecture including its tumor cells, microenvironment, and infiltrating immune cells. To this day, this is the closest human cancer model system to strengthen preclinical drug discovery and treatment decision in oncology [34,35,36,37,38]. In the past, tumor tissue slices have been used to study culture conditions and drug response assays with antineoplastic agents [8,35,39,40], analyze metastatic processes [41], and investigate new therapeutic or diagnostic strategies [42,43,44]. Other applications include studies on the regulation of tumor markers [45], the immune microenvironment [46], gene therapy [47,48], the antineoplastic potential of various agents [24,49,50], and drug sensitivity using electrochemical sensors [51], among others. Different research groups have highlighted the advantages of organotypic cultures of breast tumor tissue slices over two-dimensional cell cultures, which offers an alternative for studies that seek to individualize treatment for patients [5,40,52]. 

With regard to the latter, there are experimental tests, commercially available, that predict the individual response of a tumor to the administration of chemotherapy in colon and breast cancer. They used primary cultures obtained by enzymatic dissociation from the tumors and incubated these cells alone, or in combination with the most widely used drugs against these diseases [53,54,55]. However, we consider that the enzymatic disaggregation impedes the treatment response derived from the TME. By contrast, no proteolytic enzymes are used during the tissue slice preparation process; thus, the parenchyma remains locally intact. In addition, the 3D histological structure, cell–cell interactions, and interactions with the extracellular matrix are preserved. Therefore, it is considered that the TME also remains intact. In this work, we used organotypic cultures of breast cancer tumors cultivated in the presence of antineoplastics from the armamentarium of the Mexican Institute of Social Security to implement an experimental, low-cost methodology (the tumor sensitivity/resistance [S/R] profile) that allows optimization of current chemotherapeutic schemes, or to obtain an experimental individualized chemotherapy regimen for each patient. Our findings suggest that the tumor S/R profile can help to predict personalized therapy or optimize chemotherapeutic treatments in breast cancer.

## 2. Materials and Methods

### 2.1. Chemicals

Paclitaxel (P), epirubicin (E), 5-FU (F), cyclophosphamide (C), docetaxel (D), doxorubicin (DX), and cisplatin (CIS) were obtained from Hospital of Medical Specialties (UMAE) #25, Mexican Social Security Institute (IMSS), Monterrey, Nuevo Leon, Mexico. Insulin–transferrin–selenium (ITS) was purchased from Sigma-Aldrich (St. Louis, MO, USA). Dulbecco’s modified Eagle’s medium (DMEM)/F12 medium, fetal bovine serum, penicillin–streptomycin, and Alamar Blue^®^ (AB) were obtained from Invitrogen (Grand Island, NY, USA). Antibodies against the estrogen receptor (ER), progesterone receptor (PR), and human epidermal growth factor 2 (HER2) were obtained from Dako Agilent Technologies (Santa Clara, CA, USA). Ki 67 antibody was obtained from Santa Cruz Biotechnology (Santa Cruz, CA, USA). Reagents for general use were purchased from Sigma-Aldrich.

### 2.2. Breast Cancer Samples

After obtaining informed consent, infiltrating ductal/lobular adenocarcinoma specimens were obtained from 27 patients during therapeutic surgery at the Hospital of Gynecology and Obstetrics (UMAE #23; Monterrey, Nuevo Leon, Mexico) from IMSS. Patients who received neoadjuvant chemotherapy and/or radiotherapy were excluded from the study. Representative tumor samples were selected in situ in the surgery room by a pathologist. Fresh tissues were collected in cold sterile serum-free DMEM/F12 medium and transported at 4 °C to the laboratory for immediate processing. The interval between tumor resection, sample processing, and the start of incubation was no more than 2 h. The entire process was performed under aseptic conditions. Using the AB assay, viability was determined at 0 h for control (basal viability). The study was approved by the IMSS National Committee for Scientific Research and Ethics (Registry R-2014-785-022) and followed the international ethical standards of the Helsinki convention for research studies with human subjects. The clinical and histopathological characteristics of these patients are described in Table 1.

### 2.3. Preparation of Tumor Explants from Breast Tumors

From representative tumor samples, precision-cut tissue slices of 8–10 mm diameter and 250–300 μm thickness were prepared using a Krumdieck tissue slicer (Alabama Research & Development, Munford, AL, USA), under constant flow of Krebs-Henseleit bicarbonate buffer (KB) at 4 °C gassed with carbogen. After preparation of slices with precise thickness and diameter, 4 mm breast cancer explants were manually prepared from these slices to optimize the available tissue (scalpel and/or biopsy punch were used for this purpose). The explants were collected in KB buffer at 4 °C and subsequently placed in 6-well microplates containing DMEM/F12 medium supplemented with 10% (*v*/*v*) FBS, 1% ITS, 1 mM sodium pyruvate, 100 U/mL penicillin, 100 μg/mL streptomycin, and 25 mM glucose (supplemented DMEM/F12 medium). Plates were preincubated for 1 h at 37 °C, 5% carbon dioxide (CO_2_), with agitation at 30 rpm. Breast explants were weighted and those with similar size, form, and weight were selected. Subsequently, the explants were transferred to 24-well microplates and processed as described below.

### 2.4. Treatment of Tumor Explants with Antineoplastics

After preincubation, 5–6 explants per each antineoplastic were exposed to 20 μg/mL paclitaxel (P), 3 μg/mL epirubicin (E), 50 μg/mL 5-FU (F), 1 mg/mL cyclophosphamide (C), 20 μg/mL docetaxel (D), and 50 μg/mL cisplatin (CIS) in culture media. The P and CIS concentrations were selected according to Garcia-Chagollan, M. et al. [56] and Garcia-Davis et al. [50]. E, F, and C concentrations were selected by testing a range of doses in MCF-7 cells, and D doses were selected according to Giraud et al. [53]. The control group (100% viability) consisted of untreated explants, which were incubated only with culture media in the same conditions. The microplates were incubated for 48 h at 37 °C, 5% CO_2_, with agitation at 30 rpm. Time and culture conditions were selected as reported in previous works [49,50].

### 2.5. AB Assay for Tumor Explant Viability

The effect of the antineoplastics on the viability of the explants was determined using the metabolic AB assay (DAL1100, Invitrogen). AB is a monitor of the reducing state of living cells. The active compound is resazurin, a nonfluorescent blue dye that, when reduced by mitochondrial reductases of viable cells and tissues, is converted to resorufin, a highly fluorescent pink compound. Other cytoplasmic enzymes such as diaphorases, dihydrolipoamide dehydrogenase, nicotinamide adenine dinucleotide phosphate: quinone oxidoreductase, and flavin reductase can reduce AB [57]. After 48 h of incubation with the antineoplastics and control without treatment, the explants were washed twice with phosphate-buffered saline and incubated with 10% AB in 500 μL DMEM/F12 medium in 24-well microplates at 37 °C, 5% CO_2_, for 4 h with agitation at 40 rpm. Subsequently, 300 μL were collected from each sample and transferred to a 96-well microplate (100 μL per well). Fluorescence values were read using a multimode microplate reader (Synergy HT; BioTek Instruments, Winooski, VT, USA) at 530 nm excitation/590 nm emission wavelengths. Tissue viability is expressed as the percentage viability relative to the control, determined by calculating the percentage of AB reduction per explant as described previously by Carranza-Torres et al. [49]. Explants that showed viability values above or below 30% from the viability media value in each treatment were discarded from the analysis.

### 2.6. Histopathological Analyses

Detailed histopathologic analyses were conducted to observe changes in the histological structure and other morphologic alterations induced by the antineoplastics. The response to treatment was also evaluated considering the grade of tumor differentiation and molecular classification. After incubation with the treatments, breast cancer explants were fixed in 10% neutral formalin for 12–24 h and then embedded in paraffin using conventional histological techniques. Tissue sections (4 μm) were prepared on a microtome and mounted on glass slides. Then, the slides were deparaffinized and stained with hematoxylin and eosin. Morphological parameters analyzed included necrosis percentage, viable/damaged tumor cells, and inflammation; these were determined by two pathologists following the cancer pathological guides. In addition, the presence of tumor necrosis and the degree of histological differentiation were assessed at 0 h in tumor explants (uncultivated). The histological grade was classified into low, moderate, and poor grade according to criteria modified by Elston and Ellis [58]. Tissue viability is expressed as the percentage of viability relative to untreated controls. Tumor-infiltrating lymphocytes (TILs) were evaluated in the stroma of the explants at 0 and 48 h of incubation with the different antineoplastics following international recommendations for its quantification. The percentage of lymphocytes and plasma cell infiltration was quantified as a continuous variable, excluding areas of necrosis, artifacts, or hyaline areas [59,60]. Additionally, to estimate histological viability, a semi-quantitative assessment of necrosis was performed based on the experience and judgment of two independent pathologists. The percentage of necrosis was determined by evaluating the proportion of necrotic areas in relation to the total sample area. The observed histopathological changes included the presence of coagulative necrosis, characterized by the loss of cellular cytoplasmic integrity, contributing to the pale and uniform appearance of the tissue, with nuclei that could exhibit condensation (pyknosis) or be completely degraded. The clear transition with viable tumor tissue allowed for a precise delineation of these areas. 

### 2.7. Immunohistochemical Analyses

ER, PR, HER2, and Ki 67 expression was analyzed by immunohistochemistry on paraffin sections to assess the molecular profile of the original tumor samples. The ER, PR and Ki 67 status was evaluated using the Dako Labelled Streptavidin-Biotin2 System, Horseradish Peroxidase (LSAB2 System, HRP) methodology (Cat. #K0672), whereas HER2 expression was evaluated using the HercepTest by Dako (Cat. #K5207) and the HER2 FISH pharmDx^®^ Kit by Dako (Cat. #K5331). The procedure was performed according to the manufacturer’s recommendations. Negative and positive controls were included. The positive expression of ER and PR was assessed by the Allred method [61]. HER2 expression was evaluated following the American Society of Clinical Oncology/College of American Pathologists Clinical Practice Guidelines [62,63]. The Ki 67 expression is nuclear, and its quantification was carried out following international guidelines [64] as follows: Ki67 expression was evaluated by counting the nuclei of tumor cells stained in a specific region (200 nuclei), excluding nuclei of inflammatory cells and stroma. The proliferation index was determined as the average of the values obtained in three fields at a 40× magnification. Ki67 was analyzed as a continuous variable, and a cutoff point of interpretation greater or less than 20% was considered. After immunohistochemical analyses, samples molecular classification was determined in accordance with Alcaide-Lucena, et al. [65]. 

The stained preparations were evaluated independently by two pathologists (G-D.N.E. and E-H.O.) using a Zeiss Axiostar Plus bright-field microscope (Carl Zeiss, Dublin, CA, USA). Representative images of all treatments were obtained with the 5.0 MP Moticam camera (Motic, Kowloon City, Hong Kong, China).

### 2.8. Statistical Analyses

The present study was experimental. Statistical analyses were performed with SPSS Statistics software (version 22.0; IBM SPSS, Armonk, NY, USA). Quantitative data are expressed as the mean and standard deviation. Differences in continuous variables were analyzed by Student’s *t*-test (when there was a normal distribution), or the Mann–Whitney U test (when there was nonnormal distribution). The chi-square test was used to compare the molecular classification and the tumor S/R profile. Pearson’s correlation analysis was used to determine the association strength between the AB method and histological viability (HV). *p* < 0.05 was considered statistically significant.

## 3. Results

### 3.1. Patients

In this preliminary study, we included 27 patients; 40.7% were under 50 years old and 59.3% were older than 50 years. Obesity was the most frequent comorbidity (77.4%). Most of the patients (96.3%) did not have a family history of breast cancer; however, 63% had a family history of other types of cancer. As for their occupation, 51.8% of the patients were homemakers, 29.6% worked in an office and/or an indoor setting, and 18.6% worked in educational or health services. The main clinical and histopathological data of these patients are described in Table 1.

### 3.2. Ex Vivo Organotypic 3D Culture

**Figure 1:**
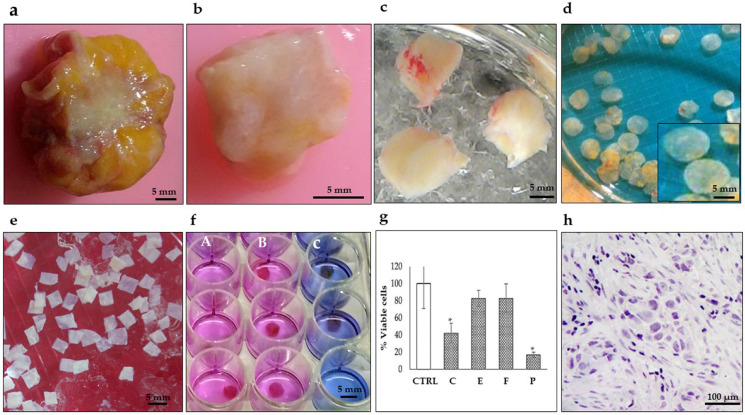
Representative photographs showing the preparation of breast tumor explants with precise thickness. (**a**) Tumor biopsy obtained during surgery. (**b**) Breast tumor after removing surrounding fat. (**c**) Tumor tissue cores (8–10 mm diameter). (**d**) Precision-cut breast tumor slices (250–300 μm thickness) prepared with a Krumdieck^®^ tissue slicer. (**e**) Breast tumor explants (4 mm diameter and 250–300 μm thickness) were manually prepared from precision cut breast tumor slices and collected in KB buffer (**f**) Breast tumor tissues after treatment culture and Alamar Blue assay. (**A**) untreated viable tissues (pink), (**B**) resistant tissues (pink) and (**C**) sensitive tissues (blue) (48 h, 37 °C, 5%CO_2_/95%O_2_, 30 rpm). (**g**) Tumor viability analysis by the Alamar Blue assay after antineoplastic treatment (C) cyclophosphamide 1 mg/mL, (E) epirubicin 3 μg/mL, (F) 5-fluororacil 50 μg/mL, and (P) paclitaxel 20 μg/mL. * *p* < 0.05 compared to the control (CTRL). (**h**) Histopathological analysis. Scale bar: (**a**–**f**), 5 mm; (**h**), 100 μm.

**Figure 2:**
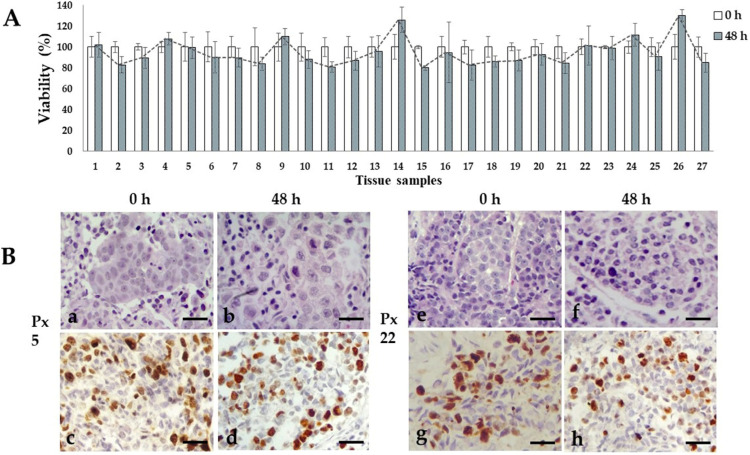
Ex vivo tissue culture viability. (**A**) Metabolic viability of untreated tissue explants after 48 h of incubation. Viability percentages remained without significant changes through the culture time. N = 27 samples by triplicate. *T* student test, *p* < 0.05 compared to viability of uncultured tissues. (**B**) Representative photographs of untreated viable invasive ductal carcinoma tissue samples from patient 5 and 22 after 48 h incubation. H&E staining (**a**,**b**,**e**,**f**) and high (>20%) Ki67 expression (**c**,**d**,**g**,**h**). Scale bar: 100 μm.

We previously reported the conditions to maintain organotypic 3D cultures of precision-cut breast cancer explants, and their application to evaluate the antineoplastic effects of natural products, and gene expression profiles of human natural killer cells [49,50,56]. In the present work, we prepared breast cancer tissue slices of 250–300 μm thickness from the 27 patients included in this study. From these precision-cut tissue slices, we obtained 30–50 explants to optimize the tumoral tissue for treatments. To obtain reproducible results and due to the limited amount of tissue, we used 5–6 explants per treatment at a single dose of each antineoplastic. Based on the availability of both explants and antineoplastic drugs, we tested 5-FU, epirubicin and paclitaxel in 26 of the 27 samples, cyclophosphamide in 24 of the 27 samples, docetaxel in 12 of the 27 samples, and doxorubicin and cisplatin in three of the 27 samples. Figure 1 shows the general sequence for the preparation, culture, treatment, and viability evaluation of breast cancer tissue explants. All 27 samples were successfully cultured; in the untreated controls, the metabolic viability (MV) analyzed by the AB assay was maintained during the 48 h incubation period (Figure 2A). Also, the histological characteristics, differentiation degree and a high proliferation index (>20% positive cells) were maintained throughout the culture (Figure 2B).

### 3.3. Necrosis and TILs in the Cultivated Explants

To analyze the response to chemotherapy in the tumor explants, the percentage of necrosis was determined after incubation with the antineoplastics (Table 2). The basal necrosis of the samples was 0–5%, and no significative necrosis was induced in culture conditions. Considering the molecular subtype, tissues with subtypes with high response to chemotherapy (triple-negative) and with low response to chemotherapy (luminal A) were analyzed. In triple-negative samples, 49.85% necrosis was found. This value almost doubled the 26.22% observed in luminal A samples, which was statistically significant (*p* = 0.014). The median basal value of TILs considering all samples was 40%, with a range of 5–85%. Regarding the molecular classification, TIL average was higher in HER2-positive and triple-negative samples, 68% and 52% respectively. In luminal A and B, however, it was 28% and 31%, respectively. No significant difference in TIL percentage was found between control and treated explants. However, a positive correlation (r = 0.038) was found between the percentage of TILs against the percentage of necrosis in the HER2-positive and triple-negative samples.

### 3.4. Sensitivity/Resistence Assay (S/R Assay)

**Figure 3:**
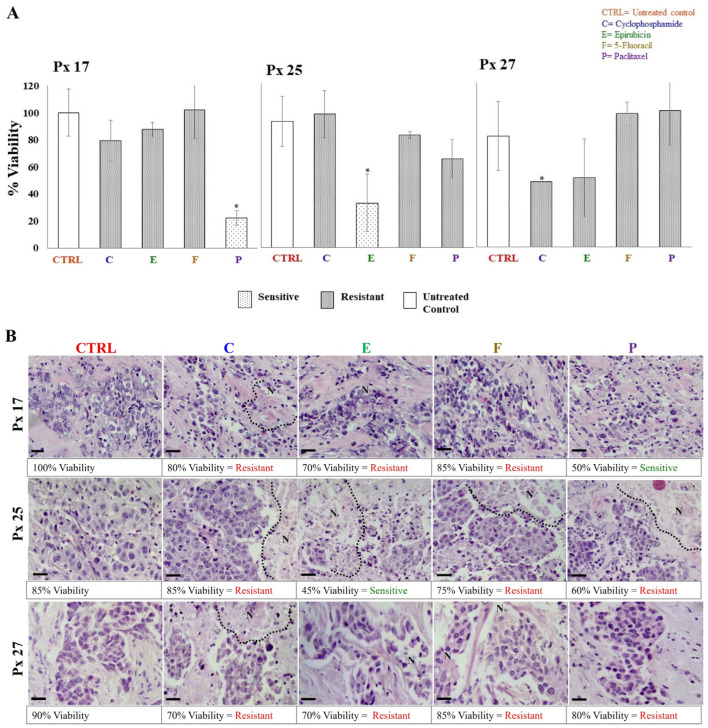
Metabolic and histological viability of explants cultivated with antineoplastics. (**A**) Resistance/sensitivity of breast tumor explants based on the Alamar Blue assay (values are expressed as mean ± standard deviation, n = 3–5 explants per treatment, * *p* < 0.05 compared to the control). (**B**) Structural viability by histopathological analysis. Necrotic areas are delimited by dotted lines. Values are presented as the average of 5–6 explants per treatment. Tumor viability was evaluated 48 h after treatment with 1 mg/mL cyclophosphamide (C), 3 μg/mL epirubicin (E), 50 μg/mL 5-fluororacil (F), and 20 μg/mL paclitaxel (P). Scale bar: 100 μm.

The S/R of breast cancer explants to each antineoplastic was evaluated after 48 h of incubation through metabolic viability (MV) by the AB assay, and histological viability (HV) by histopathological analyses. In both analyses, tumor samples were considered sensitive if the tissue showed viability values ≤ 50%, and resistant when viability was >51%. The tumor S/R profile for each patient was obtained by comparing data obtained by MV and HV. As expected, 27 different tumor S/R profiles were obtained, one for each patient. In 26 of the profiles, we were able to test four or more antineoplastics (Table 3). As representative examples, the viability results of patients 17, 25, and 27 analyzed from the metabolic and histological perspectives are shown in Figure 3A,B, respectively. In these cases, tumor explants were independently incubated with cyclophosphamide, epirubicin, 5-FU, and paclitaxel. On the one hand, Figure 3A shows that the tumor explants of patients 17 and 25 were sensitive to paclitaxel (22.2% viability) and epirubicin (35.2% viability), respectively, but showed resistance to the other antineoplastics (viability > 70.4%). On the other hand, patient 27 was resistant to all treatments (viability of 52–100%). For HV, the structural conservation, necrosis and inflammation percentages, tumor differentiation degree, and presence of connective tissue were considered. The percentages of HV for each antineoplastic were assigned considering the S/R criteria mentioned above. Figure 3B shows representative images of the histopathological analyses from the same patients as Figure 3A. Similar to MV findings, explants from patients 17 and 25 showed sensitivity to paclitaxel (50% viability) and epirubicin (45% viability), respectively, and resistance (>60% viability) to the other treatments. Also, explants from patient 27 showed resistance to the four antineoplastic (75–85% viability). In all cases, we observed preserved cell structures, the presence of fibrous tissue, erythrocytes, and inflammatory infiltrate, and cell death areas. When treatments showed differences between results obtained by HV and MV, we considered these cases to have intermediate sensitivity. We found the greatest number of these differences for treatment with taxanes. For all patients’ samples, we analyzed the viability values of MV and HV by Pearson’s correlation analysis. A direct correlation for cyclophosphamide (r = 0.007) and epirubicin treatments (r = 0.029) was found. Table 3 shows a summary of all tumor S/R profiles.

Overall, 88.5% of samples were resistant to 5-FU, 78.4% to anthracyclines, 66.7% to cyclophosphamide, 66.7% to docetaxel, and 16% to paclitaxel. When the relationship between resistance to cyclophosphamide and hormonal status was analyzed, we found a significant difference in 83.3% of the hormone receptor-positive tumor samples luminal A and B (*p* = 0.027). However, there were no significant differences between resistance to the other antineoplastics regarding molecular classification and histological grade.

## 4. Discussion

Despite the recent and remarkable advances in the treatment of breast cancer, as well the evolution toward personalized therapy [7], it has not been possible to effectively foretell the response of patients to therapeutic treatments [40]. In this study, we used precision-cut slices/explants of breast tumor tissue to obtain tumor S/R profiles as an experimental tool to predict and individualize chemotherapy treatment for patients with breast cancer.

It is important to take into consideration the organotypic culture technique, proper tissue handling; the time elapsed from in situ collection, transport, processing; and the start of incubation in order to reduce necrosis induced by mechanical damage during the tissue slicing process and manipulation, to ensure that the tissue remains viable [31] and to abate variations in the results [24]. Taking this in consideration, in this study, the processing time from when the tissue sample was acquired to its final incubation period was less than 2 h. Usually, it is possible to obtain slices with adequate thickness and size; however, the size of the tumor fragment provided by the pathologists, as well as its consistency (e.g., soft, firm, fibrotic, or with necrotic zones), are also very important because the quality of the tumor slices depends directly on these factors. To optimize the human samples, tissue tumor explants with uniform thickness were prepared from precision-cut tumor slices. Based on our experience, we made the necessary adjustments for each sample, as reported previously [24,32,52,66].

The histological, cytological characteristics, as well as the degree of differentiation of the tumor were perpetuated during the ex vivo culture, similar to that observed by different researchers using other tumor tissue slice models [31,35,67]. This study also observes the high proliferation rate during the culture period (Figure 2B), which is consistent with previous reports [68,69], suggesting that both the selected time and the culture conditions are suitable for conducting assays to assess the effects of antineoplastic agents on the tissues. To obtain sufficient and reliable results that allow the tumor S/R profile to be considered as a preclinical model, it is necessary to select and carry out drug response tests with their respective controls [70] and to acknowledge the mode of action of each drug or compound that is evaluated [40]. We contemplated these factors evaluating the metabolic viability (MV) using the AB assay, which is a well-known and widely accepted method for this purpose [70,71,72]. We compared the results obtained by this assay (MV) with the histologic viability (HV), which allows the microscopic observation of viable cells and patterns of cell death in tissues. We take into account the HV criteria due to the difficulties that can arise in interpreting assays based on mitochondrial activity and adenosine triphosphate (ATP) levels, among other factors [31]. Although there are other methods that could be more accurate [24,35,73], they tend to increase economical costs due to the need for more complex analyses, infrastructure, and supplies, which are often unaffordable for public health systems, especially in developing countries.

We observed discrepancies between the results obtained by MV and HV in 35.1% of the explants treated with taxanes (i.e., docetaxel and paclitaxel). This finding may have been caused due to its primary mechanism of action which lies in stabilizing microtubules to inhibit the cell cycle in the M phase, and, consequently, causes cell death by mitotic arrest [74], which would be its secondary mechanism of action as a result to exposure for more than 48 h [75]. While we were able to observe the effects on HV and MV in explants sensitive to paclitaxel, in explants classified as resistant and/or with intermediate sensitivity, there was no effect based on HV, where the integrity of the membranes was maintained, and the percentage of cell death was not altered significantly during the 48 h treatment period. Ladan et al. [40] addressed this technical difficulty by measuring cell blockage of mitosis rather than cell death or proliferation to assess the effects of docetaxel on cells, because taxanes inhibit cell growth by a different mechanism than that of chemotherapy agents, which act by damaging DNA. For this reason, we evaluated the mitotic index as an additional test to establish whether there was S/R to paclitaxel and/or docetaxel. The relative value of the mitotic index was not influenced by the 48 h treatment in any of the explants with resistance or intermediate sensitivity to those compared to control untreated explants.

Nonetheless, it is important to mention that the decrease in MV may be due to the direct effects of taxanes because they reduce cellular respiration through conformational changes in mitochondrial protein complexes such as ATP synthase (complex V), prohibitins, and voltage-gated anion channel proteins [74]. An advantage of this organotypic culture system is that it allows sectioning tumor fragments of at least 1 cm^2^ to produce precision-cut tumor slices (10 mm diameter and 250–300 μm thickness), each of these slices produces 3–4 explants from different areas of the tumor. This approach optimizes the use of available tissue and ensures that all different tumor regions are included, which could feasibly represent the complex heterogeneity of each tumor [31]. This could also lead to variable results due to the presence of different cellular components of the TME within each unique explant sample. The relevance of the tumor stroma in preclinical models is undeniably vital [31]. As previously proven by Gerlinger et al. [25] and Kenerson et al. [76], which demonstrated different intratumoral zones that were separated only by a few millimeters and had different metabolic activities and gene expression profiles, which reflect real tumor behavior due in part to this heterogeneity. Ex vivo 3D models like the one we describe in this work allows the inclusion of different inter- and intratumor regions that constitute the microenvironment of each tumor, which in turn emphasizes the influences in the selection of optimal treatment for each patient [39,66,76,77] and provides a closer view of what happens in the niche of cancer cells in vivo.

Furthermore, the most frequently reported molecular subtype of breast cancer is luminal A [7], which concurs with our results (frequency of 59.3%). The fact that we found no significant differences between the response to the tested antineoplastic regarding the molecular classification and histological grade may be due to the small number of samples of each molecular subtype. However, most luminal A tissues were resistant to the treatments, except paclitaxel. These results are similar to the low response reported for this drug because the treatment for luminal A breast cancer tumors is hormonal therapy [7], which was not evaluated in the present study.

To establish a relationship between the viability of the tumor explants cultivated ex vivo and the in vivo response, the percentage of necrosis as a proxy to the pathological response was determined, considering that triple-negative tumors have a higher rate of response, while luminal A tumors have a lower response rate [78,79,80]. The percentage of necrosis in response to the treatments in triple-negative samples was 49.85%, and 26.22% in luminal A; the difference was statistically significant (*p* < 0.05). These values are very similar to those reported for pathologic complete response rates in these molecular subtypes [81]. As for inflammation, in control and cultivated explants, no significant difference in TIL percentage was found, which was reasonably due to the sample size.

Although further validation is required, the results suggest that the model we used here agrees with the expected clinical response [79,80]. This is in accordance with the findings reported by Ruvalcaba-Limón et al. [79], who found a weak negative correlation between the percentage of ERs and the clinical response in primary tumors. Similarly, Giménez-Martínez et al. [82] reported high chemosensitivity in terms of complete pathological response in tumors with negative hormone receptors and low response rate in luminal tumors. This is important if we consider that our proposal seeks to have an impact in countries with limited funding resources, where molecular tests are not economically viable in public hospitals. In addition, the results could also be useful to identify luminal A patients, with poor or no responses, to find alternatives to chemotherapy, or reduce the burden of costs in public health systems.

This ex vivo assay could be applied in any third level hospital, with minimum funding in the clinical laboratory, oncology, and pathology departments, because it allows testing on tumoral tissues using the same drugs from the hospital’s already in stock treatments that are used for breast cancer. This would allow the selection of the best optimal treatment, prevent undesirable side effects, and lower expenditure to the health care system, reducing the cost of expensive genomic profiles that assess the utility of chemotherapy. In addition, the genomic profiling is validated in patients with early-stage breast cancer up to N1 (1–3 lymph nodes), and the tumor S/R profile can also be used in advanced cancers, such as the ones present in this work. Properly distributing medical spending and the use of available assets is a preeminent health issue in developing countries, where resources are limited. Therefore, analyses of the tumor S/R profile represent a useful tool that is easy to apply in a public health system. The results can be obtained in a minimum of one to two weeks and can be used to provide target therapies and improve the success rate of chemotherapy.

## 5. Conclusions

Compared to other models, the tumor S/R profile obtained while culturing representative samples of tumors from each patient provides results in less than 2 weeks, is reproducible, low cost, and could help predict personalized chemotherapy or optimize the treatment for hormone-sensitive patients with breast cancer, avoiding the use of ineffective antineoplastic agents and their toxic effects and, in turn, reducing the system costs of health. Its clinical utility will depend on the results of future clinical studies in which the patient receives its therapeutic treatment based on their tumor S/R profile while being closely monitored by clinical oncologists to assess their evolution and response to treatment.

## Figures and Tables

**Table 1:** Patient clinical and histopathological characteristics.

Patient	Age (yr.)	Histologic Type	Tumor Size (cm)	Birads	Stage	Tumor Grade	Estrogen Receptor (ER)	Progesterone Receptor (PR)	Her2 Status	Molecular Subtype
1	49	mixed ductal and lobular	3.5	5	IIIA	G2	+	+	−	Luminal A
2	70	invasive ductal	14	4	IIIB	G2	+	+	−	Luminal A
3	35	invasive ductal	3	4	IIB	G3	−	−	−	Triple Negative
4	81	invasive lobular	7	5	IIB	G2	+	+	−	Luminal A
5	52	invasive ductal	6	4	IIIA	G3	−	−	+	HER2
6	60	invasive ductal	5.5	4	IIIB	G2	−	−	−	Triple Negative
7	36	invasive ductal	4.5	4	IIIB	G3	+	+	+	Luminal B
8	29	invasive ductal	4	3	IIB	G2	+	−	+	Luminal B
9	46	invasive ductal	6	5	IIIA	G3	+	−	+	Luminal B
10	69	invasive ductal	5	5	IIIA	G3	+	+	−	Luminal A
11	63	invasive ductal	2.5	5	IIB	G2	−	−	+	HER2
12	74	invasive ductal	4	4	IIB	G2	+	+	−	Luminal A
13	46	invasive ductal	3	5	IIIA	G2	−	−	−	Triple Negative
14	49	invasive ductal	4	5	IIIA	G2	+	+	−	Luminal A
15	41	invasive ductal	3.5	4	IIIC	G3	+	+	−	Luminal A
16	59	invasive ductal	7.5	4	IIIB	G2	+	+	−	Luminal A
17	72	invasive lobular	4	5	IIIA	G3	+	+	−	Luminal A
18	65	invasive ductal	5.5	4	IIA	G2	−	−	−	Triple Negative
19	50	mixed ductal and lobular	4.5	4	IIIA	G3	−	−	−	Triple Negative
20	69	invasive ductal	6.5	4	IIIC	G2	+	+	−	Luminal A
21	45	invasive ductal	6	5	IIIA	G2	+	+	−	Luminal A
22	87	invasive ductal	9	5	IIA	G2	+	−	−	Luminal A
23	41	invasive ductal	7.5	5	IIIA	G3	+	−	−	Luminal A
24	82	invasive ductal	6	4	IIIA	G2	+	+	−	Luminal A
25	56	invasive ductal	6	5	IIIB	G2	+	+	−	Luminal A
26	54	invasive ductal	5	4	IIIB	G2	+	+	−	Luminal A
27	46	invasive ductal	4	4	IIIA	G2	−	−	+	HER2

**Table 2:** Necrosis in triple-negative and luminal A cultivated explants.

**TRIPLE- NEGATIVE**								
	**Antineoplastic**							
**Px**	**C**	**E**	**F**	**P**	**D**	**DX**		**Average**
3	20%	10%	60%	30%	-	-		30%
6	60%	60%	20%	50%	40%	30%		43.33%
13	70%	40%	70%	70%	-	-		62.5%
18	30%	20%	30%	20%	-	-		25%
19	95%	90%	85%	85%	-	-		88.75% (90% basal)
**General Average**								**49.85%**
**LUMINAL A**								
	**Antineoplastic**							
**Px**	**C**	**E**	**F**	**P**	**D**	**DX**	**CIS**	**Average**
1	-	20%	40%	60%	15%	-	-	33.75%
2	-	0%	0%	20%	0%	-	-	5%
4	60%	50%	35%	40%	40%	-	-	45%
10	5%	20%	10%	10%	10%	-	-	11%
12	15%	10%	15%	15%	-	-	-	13.75%
14	25%	30%	30%	40%	-	-	-	31.25%
15	40%	15%	20%	20%	10%	-	-	21%
16	10%	40%	20%	20%	20%	-	-	22%
17	20%	30%	15%	40%	-	-	-	26.25%
20	20%	35%	-	75%	-	-	-	43.33%
21	40%	35%	10%	55%	-	-	-	35%
22	10%	5%	5%	5%	-	-	-	6.25%
23	30%	10%	10%	15%	-	-	-	16.25%
24	20%	20%	20%	40%	-	-	-	25%
25	15%	30%	25%	40%	-	-	70%	36%
26	0%	90%	10%	-	-	-	95%	48.75%
**General Average**								**26.22%**

Necrosis percentages after treatment with (C) cyclophosphamide 1 mg/mL, (D) docetaxel 20 μg/mL, (E) epirubicin 3 μg/mL, (F) 5-fluororacil 50 μg/mL, (P) paclitaxel 20 μg/mL, (DX) doxorubicin 3 μg/mL, and (CIS) cisplatin 50 μg/mL.

**Table 3:** Patient Tumor S/R Profile (T S/R P).

		ANTINEOPLASTIC								ANTINEOPLASTIC					
		Taxanes		Anthracyclines		Fluoropyrimidine	Alkylating Agents			Taxanes		Anthracyclines	Fluoropyrimidine	Alkylating Agents	
Px		D	P	E	DX	F	C	Px		D	P	E	F	C	CIS
1	MV	R	S	R	-	R	-	15	MV	R	S	R	R	R	-
	HV	R	S	R	-	R	-		HV	R	S	R	R	R	-
	**T S/R P**	R	S	R	-	R	-		**T S/R P**	R	S	R	R	R	-
2	MV	R	S	R	-	R	-	16	MV	R	R	R	R	R	-
	HV	R	S	R	-	R	-		HV	R	S	R	R	R	-
	**T S/R P**	R	S	R	-	R	-		**T S/R P**	R	I	R	R	R	-
3	MV	R	S	R	-	R	-	17	MV	-	S	R	R	R	-
	HV	R	R	R	-	R	-		HV	-	S	R	R	R	-
	**T S/R P**	R	I	R	-	R	-		**T S/R P**	-	S	R	R	R	-
4	MV	R	R	R	-	R	R	18	MV	-	S	S	R	S	-
	HV	R	R	R	-	R	S		HV	-	S	R	R	S	-
	**T S/R P**	R	R	R	-	R	I		**T S/R P**	-	S	I	R	S	-
5	MV	R	S	R	R	R	R	19	MV	-	S	S	R	S	-
	HV	R	R	R	R	R	R		HV	-	S	S	S	S	-
	**T S/R P**	R	I	R	R	R	R		**T S/R P**	-	S	S	I	S	-
6	MV	S	R	S	R	R	S	20	MV	-	S	R	R	R	-
	HV	R	-	S	R	R	S		HV	-	S	R	-	R	-
	**T S/R P**	I	ND ^#^	S	R	R	S		**T S/R P**	-	S	R	ND ^#^	R	-
7	MV	S	S	R	R	R	R	21	MV	-	S	R	R	R	-
	HV	R	S	S	R	S	S		HV	-	S	R	R	R	-
	**T S/R P**	I	S	I	R	I	I		**T S/R P**	-	S	R	R	R	-
8	MV	R	S	-	-	R	R	22	MV	-	R	R	R	R	-
	HV	R	R	-	-	R	R		HV	-	R	R	R	R	-
	**T S/R P**	R	I	-	-	R	R		**T S/R P**	-	R	R	R	R	-
9	MV	S	S	R	-	R	S	23	MV	-	S	R	R	R	-
	HV	R	R	R	-	R	S		HV	-	R	R	R	R	-
	**T S/R P**	I	I	R	-	R	S		**T S/R P**	-	I	R	R	R	-
10	MV	S	S	R	-	R	R	24	MV	-	S	R	R	R	-
	HV	R	R	R	-	R	R		HV	-	S	R	R	R	-
	**T S/R P**	I	I	R	-	R	R		**T S/R P**	-	S	R	R	R	-
11	MV	-	S	R	-	R	S	25	MV	-	R	S	R	R	S
	HV	-	S	S	-	S	S		HV	-	R	S	R	R	S
	**T S/R P**	-	S	I	-	I	S		**T S/R P**	-	R	S	R	R	S
12	MV	-	S	R	-	R	R	26	MV	-	R	S	R	R	S
	HV	-	R	R	-	R	R		HV	-	R	S	R	R	S
	**T S/R P**	-	I	R	-	R	R		**T S/R P**	-	R	S	R	R	S
13	MV	-	S	R	-	R	S	27	MV	-	R	R	R	R	R
	HV	-	S	R	-	R	S		HV	-	R	R	R	R	R
	**T S/R P**	-	S	R	-	R	S		**T S/R P**	-	R	R	R	R	R
14	MV	-	S	R	-	R	R								
	HV	-	R	R	-	R	R								
	**T S/R P**	-	I	R	-	R	R								

Determination of individual tumor S/R profile (T S/R P) after treatment with 1 mg/mL cyclophosphamide (C), 20 μg/mL docetaxel (D), 3 μg/mL epirubicin (E), 50 μg/mL 5-fluororacil (F), 20 μg/mL paclitaxel (P), 3μg/mL doxorubicin (DX), and 50 μg/mL cisplatin (CIS). MV: Metabolic viability, HV: Histological viability, R: Resistant (red), I: Intermediate (yellow), S: Sensitive (green). # Not enough discrimination elements.

## Data Availability

The data presented in this study are available in this article.
